# Joint Working with Integrated Care Boards to Reduce Out of Area Rehabilitation Patient Numbers, Improve Quality of Care and Have Clear Interventions

**DOI:** 10.1192/bjo.2026.11355

**Published:** 2026-06-30

**Authors:** Mike Bourke

**Affiliations:** Cheshire and Wirral Partnership NHS Foundation Trust, Cheshire, United Kingdom

## Abstract

**Aims:**

CQC (2018, 2019) is concerned about the high numbers of patients situated a long way from home which could result in social detachment and cut off from the local services that will provide care following discharge. Cheshire and Wirral Partnership NHS Foundation Trust was identified by CQC (2018) as being in the top twenty NHS mental health trusts with the highest number of patients placed in a mental health rehabilitation ward funded by a different provider. Rehab case management provides key worker responsibility, utilising expertise and specialist skills for patients in hospitals in line with NHS England commissioner guidance (2024) to improve quality and ensure services are effective, evidence based and safe. Thus, weekly multi-disciplinary team (MDT) meetings with rehab case management and Cheshire and Wirral Integrated Care Boards (ICBs) were set up in August 2024 to monitor the key components of the admission and discharge planning.

**Methods:**

Community mental health transformation is at the heart of the approach where a guiding principle of the rehab case management team is community based by default, inpatient by exception. A data analysis review was undertaken through comparing the results of pre-and post-setting up the MDT with a focus on purpose of admission, defined therapeutic interventions and estimated date of discharge. An admission should only be as long as necessary with emphasis on improved reporting of patients who are clinically readyfor discharge (CRFD). Rehab case management fosters shared-decision making through patient-reported outcome measures including DIALOG and Goal-Based Outcomes to monitor progress, guide treatment and facilitate discharge planning. The Friends and Family Test offers a mechanism for evaluating patient experience and improving quality.

**Results:**

The review demonstratedeffectiveness of collaborative working between rehab case management and Cheshire and Wirral ICBs to reduce the number of patients in out of area hospitals. Improvement in mental health rehabilitation pathways is gleaned through the lens of ‘in sight and in mind’. There have been 14 patients from Cheshire discharged and 14 patients discharged since the inception of the MDT.

**Conclusion:**

The findings suggest that the collaborative working with ICBs has significantly helped improve patient flow, reduce discharge delays and provided a structured, data-driven approach to managing inpatient stays. It is this focus that has achieved the objectives of the NHS England commissioner guidance and the NHS 10 Year Health Plan for England to reduce the reliance on inpatient care and deliver interventions in the least restrictive setting.

